# Midwives' Registration Bill

**Published:** 1898-05-14

**Authors:** 


### MIDWIVES' REGISTRATION BILL.

MISS ROSALIND PAGET (certificated midwife), Hon. Treasurer, Midvives' Institute, writes: I shall be very grateful if you will allow me, as the representative of the Incorporated Midwives' Institute on the Midwives' Bill Committee, to say a word with regard to part of Mrs. Garrett Anderson's letter to the *Times* of April 30th. It is always dangerous to find motives for other people, and in this instance Mrs. Garrett Anderson has been betrayed into finding a motive for the promoters of the Midwives' Bill, which, to those who know the circumstances under which it was drafted, is simply ludicrous. She states that it is the outcome of the desire on the part of the midwives for complete independence and for a position of professional dignity. She further says that the question of the position of midwives "lies at the core of the whole matter," and she makes the entirely unfounded statement that it is "the true basis on which the agitation for registration on the part of the promoters rests."

May I lay before you what the position of the Incorporated Midwives' Institute really is? We, its members, have voluntarily prepared ourselves in the best way at present attainable for an occupation which, in the present state of the law, may, however, be followed without preparation or qualification by anyone. Our institute approves of the Bill now before Parliament, because it believes it to be as just and fair to the midwife as is compatible with the adequate protection of the parturient woman and with a due regard to the susceptibilities and interests of the general medical practitioner. Does Mrs. Anderson seriously believe that this Bill is being promoted in the interests of the midwife. I will not be impolite enough to suggest that she has not thoroughly mastered the contents of a measure on which she writes at such length, but in what part of it does she find this complete independence for the midwife—this position of professional dignity? In this Bill we midwives are placed under the control of the Midwives' Board, on which sit twelve registered medical practitioners and six nominees of the Privy Council, who may also be doctors. This board is practically absolute; it can restrict our practice, suspend us from our work, and even remove our names from the register. Besides this, we are to be supervised by the Local Sanitary Authority. I fail to see where "the complete independence" comes in. At present, in the absence of any legislation, the only check to our independence is the coroner.

In return for submitting to this discipline, we obtain in the proposed Bill (1) the protection of the title midwife, which is, in our opinion, an undoubted advantage; (2) after years of persistence on the part of the Midwives' Institute the right to nominate three of the twelve medical practitioners on the Midwives' Board. We do not call this a position of much "professional dignity," whatever Mrs. Garrett Anderson may think. The fear of the competition of the midwife expressed by Mrs. Garrett Anderson is exceedingly flattering, but, from our point of view, a little comical. The question is entirely one of supply and demand. The public will choose the attendant it prefers, quite irrespectively of the rights or sentiments of either doctor or midwife, and this we had all better recognise at once.

With regard to the totally unjustifiable suggestion on the part of Mrs. Garrett Anderson as to the probable effect of this Bill on public morals, does she really believe that we should necessarily undergo moral deterioration in acquiring that minimum of knowledge which will enable us to mitigate the sufferings of members of our own sex, and even if she does, is she of opinion that the control and supervision by the twelve medical members of the Board will be useless to prevent midwives sinning in this particular? I can only remind her that, after all, the public has a very sufficient safeguard with regard to midwives that it has not, for instance, with regard to the registered medical practitioner, for we cannot sign death certificates, and so our misdeeds would sooner or later be brought to light. In conclusion, may I say that this letter is only intended as an answer to Mrs. Garrett Anderson's attack on the trained midwife.

"Fairplay," whose letter was received after the above was in type, will see that the matter is dealt with by Miss Paget.

### CONVALESCENT HOME AT EAST FINCHLEY.

Mr. R. Langton Cole writes: As architect of the Convalescent Home at East Finchley, described in your last issue, will you allow me to point out that the home was opened on June 16th (not January); that the foul linen bin is below the window shown near it, so that the lobby is fully ventilated; that one of the store-rooms upstairs has a skylight; and that the lavatory opposite the entrance is for the use of the doctor, visitors, &c.

